# The LIFE TRIAD of emergency general surgery

**DOI:** 10.1186/s13017-022-00447-7

**Published:** 2022-07-25

**Authors:** Federico Coccolini, Massimo Sartelli, Yoram Kluger, Aleksei Osipov, Yunfeng Cui, Solomon Gurmu Beka, Andrew Kirkpatrick, Ibrahima Sall, Ernest E. Moore, Walter L. Biffl, Andrey Litvin, Michele Pisano, Stefano Magnone, Edoardo Picetti, Nicola de Angelis, Philip Stahel, Luca Ansaloni, Edward Tan, Fikri Abu-Zidan, Marco Ceresoli, Andreas Hecker, Osvaldo Chiara, Gabriele Sganga, Vladimir Khokha, Salomone di Saverio, Boris Sakakushev, Giampiero Campanelli, Gustavo Fraga, Imtiaz Wani, Richard ten Broek, Enrico Cicuttin, Camilla Cremonini, Dario Tartaglia, Kjetil Soreide, Joseph Galante, Marc de Moya, Kaoru Koike, Belinda De Simone, Zsolt Balogh, Francesco Amico, Vishal Shelat, Emmanouil Pikoulis, Isidoro Di Carlo, Luigi Bonavina, Ari Leppaniemi, Ingo Marzi, Rao Ivatury, Jim Khan, Ronald V. Maier, Timothy C. Hardcastle, Arda Isik, Mauro Podda, Matti Tolonen, Kemal Rasa, Pradeep H. Navsaria, Zaza Demetrashvili, Antonio Tarasconi, Paolo Carcoforo, Maria Grazia Sibilla, Gian Luca Baiocchi, Nikolaos Pararas, Dieter Weber, Massimo Chiarugi, Fausto Catena

**Affiliations:** 1 General, Emergency and Trauma Surgery, Pisa University Hospital, Via Paradisia 1, 56100 Pisa, Italy; 2 General and Emergency Surgery, Macerata Hospital, Macerata, Italy; 3 Division of General Surgery, Rambam Health Care Campus, Haifa, Israel; 4 Emergency Surgery, Emergency Surgery of the Research Institute of Emergency Medicine Named After I.I. Dzhanelidze, St. Petersburg, Russia; 5 Department of Surgery, Tianjin Nankai Hospital, Nankai Clinical School of Medicine, Tianjin Medical University, Tianjin, China; 6 General Surgery, Ethiopian Air Force Hospital, Bishoftu, Oromia Ethiopia; 7 General, Acute Care, Abdominal Wall Reconstruction, and Trauma Surgery Foothills Medical Centre, Calgary, AB Canada; 8 Department of General Surgery, Hôpital Principal de Dakar Military Teaching Hospital, Dakar, Sénégal; 9 Ernest E. Moore Shock Trauma Center at Denver Health, Denver, CO USA; 10 Trauma Surgery Department, Scripps Memorial Hospital, La Jolla, CA USA; 11 Department of Surgical Disciplines, Immanuel Kant Baltic Federal University, Regional Clinical Hospital, Kaliningrad, Russia; 12 Emergency and Trauma Surgery, Papa Giovanni XXIII Hospital, Bergamo, Italy; 13 ICU Department, Maggiore Hospital, Parma, Italy; 14 Unit of General Surgery, CARE Department Henri Mondor University Hospital (AP-HP), Faculty of Medicine, University of Paris Est, UPEC, Créteil, France; 15 College of Osteopathic Medicine, Rocky Vista University, Parker, CO 80134 USA; 16 General Surgery, Pavia University Hospital, Pavia, Italy; 17 Emergency Medicine Department, Radboud Universitair Medisch Centrum, Nijmegen, The Netherlands; 18 Department of Surgery, College of Medicine and Health Sciences, UAE University, Al-Ain, UAE; 19 General Surgery Department, Monza University Hospital, Monza, Italy; 20 Department of General and Thoracic Surgery, University Hospital of Giessen, Giessen, Germany; 21 Emergency and Trauma Surgery, Niguarda Hospital, Milan, Italy; 22 Emergency and Trauma Surgery, Gemelli University Hospital, Rome, Italy; 23 General Surgery Department, Mozyr Hospital, Mozyr, Belarus; 24 ASUR Marche, AV5, Hospital of San Benedetto del Tronto, San Benedetto del Tronto, Italy; 25 Dipartimento Di Chirurgia Generale E Specialistica “Paride Stefanini”, La Sapienza University of Rome, Rome, Italy; 26 General Surgery Department, Plovdiv University Hospital, Plovdiv, Bulgaria; 27 General Surgery Department, Insubria University, Varese, Italy; 28 General Surgery Department, Campinas University, Campinas, Brazil; 29 Department of Minimal Access and General Surgery, Government Gousia Hospital, Srinagar, Jammu and Kashmir India; 30 Surgery Department, Radboud University Medical Center, Nijmegen, The Netherlands; 31 Department of Gastrointestinal Surgery, Stavanger University Hospital, Stavanger, Norway; 32 Trauma Department, University of California, Davis, Sacramento, CA USA; 33 Trauma/Acute Care Surgery, Department of Surgery, Medical College of Wisconsin, Milwaukee, WI USA; 34 Department of Traumatology and Critical Care Medicine, National Hospital Organization Kyoto Medical Center, Kyoto, Japan; 35 Visceral and Metabolic Minimally Invasive Surgery, Centre Hospitalier Intercommunal de Poissy/Saint Germain en Laye, Saint Germain en Laye, France; 36 Department of Traumatology, John Hunter Hospital and University of Newcastle, Newcastle, NSW Australia; 37 HPB Surgery, Tan Tock Seng Hospital, Tan Tock Seng, Singapore; 38 General Surgery, Hospital, National and Kapodistrian University of Athens (NKUA), Athens, Greece; 39 Department of Surgical Sciences and Advanced Technologies, University of Catania, Catania, Italy; 40 General Surgery, San Donato Hospital, Milan, Italy; 41 Helsinki University Hospital and University of Helsinki, Helsinki, Finland; 42 Department of Trauma, Hand-, and Reconstructive Surgery, University Hospital Frankfurt, Goethe-University, Frankfurt am Main, Germany; 43 Department of Surgery, Virginia Commonwealth University School of Medicine, Richmond, VA USA; 44 University of Portsmouth UK & Portsmouth Hospitals University NHS Trust UK, Portsmouth, UK; 45 Department of Surgery, University of Washington School of Medicine, Harborview Medical Center, Seattle, USA; 46 Department of Surgery, Nelson R. Mandela School of Clinical Medicine, University of KwaZulu-Natal, Durban, South Africa; 47 Trauma and Burns Service, Inkosi Albert Luthuli Central Hospital, Department of Health, KwaZulu-Natal, Mayville, Durban, South Africa; 48 Division of General Surgery, School of Medicine, Istanbul Medeniyet University, Kadıkoy/Istambul, Turkey; 49 Department of Surgical Science, University of Cagliari, Cagliari, Italy; 50 HUS Abdominal Center, Emergency Surgery, Meilahti Tower Hospital, Helsinki, Finland; 51 Department of General Surgery, Anadolu Medical Center, Kocaeli, Turkey; 52 Trauma Center, Groote Schuur Hospital and University of Cape Town, Cape Town, South Africa; 53 General Surgery, Tbilisi State Medical University, Tbilisi, Georgia; 54 Emergency Surgery Department, Parma University Hospital, Parma, Italy; 55 Department of Morphology, Surgery and Experimental Medicine, University Hospital of Ferrara and University of Ferrara, Ferrara, Italy; 56 General Surgery, Department of Clinical and Experimental Sciences, University of Brescia, Brescia, Italy; 57 Department of General Surgery, Royal Perth Hospital, Division of Surgery, School of Medicine, The University of Western Australia, Perth, Australia; 58 Emergency and Trauma Surgery, Bufalini Hospital, Cesena, Italy

**Keywords:** Emergency General Surgery, Formation, Data, Outcomes, Effectiveness, Learning, Planning

## Abstract

Emergency General Surgery (EGS) was identified as multidisciplinary surgery performed for traumatic and non-traumatic acute conditions during the same admission in the hospital by general emergency surgeons and other specialists. It is the most diffused surgical discipline in the world. To live and grow strong EGS necessitates three fundamental parts: emergency and elective continuous surgical practice, evidence generation through clinical registries and data accrual, and indications and guidelines production: the LIFE TRIAD.

## Background

Emergency General Surgery (EGS) is a surgical discipline encompassing all traumatic and non-traumatic surgical emergencies. EGS is the most diffuse practiced surgical discipline in the entire world. Almost all general surgeons deal daily with surgical emergencies. The emergency general surgeon can be currently considered as the last surgeon who is able to manage surgical emergencies in almost every body region within an emergency setting including traumatic and non-traumatic conditions. This entails taking critical and serious decisions that cannot be reversed within a short time and may affect life. It requires a special leading personality that has the adequate knowledge, skills, professionalism, and critical reasoning to achieve this highly demanding task. In fact, the acuity of the patients admitted for acute surgical diseases is unique and deserves special attention [1]. Recent studies have shown that EGS patients are at a uniquely higher risk for complications following surgery, with EGS patients up to eight times more likely to die compared to patients undergoing the same procedure electively. Approximately half of all patients undergoing EGS will have a postoperative complication [2, 3]. The emergency general surgeon is the one trained to manage together both surgical and physiological derangements of such complicated patients, especially in an era when patients are becoming older and older. Some aspects, however, must be analyzed and communicated about this diffused and underestimated discipline. Notwithstanding the EGS importance and diffusion, however, it is astonishingly an orphan specialty. In some places, acute care surgery concept is diffused, but it is different from EGS. Acute care has been developed and diffused within some countries. It encompasses several skills and a specific training that cannot be applied universally due to its specificity for the healthcare systems in which it was originally developed. In fact, in most part of the countries, EGS is differently distributed among the different actors where the general surgeon is the pivot around which the system moves and ICU doctors and orthopedics/traumatologists are differently included. This is even more true in referral or hub hospitals where the most severe emergencies are centralized, and the patients’ flux is more represented. In low–middle-income countries, EGS is the first surgical need to achieve in rural or city hospitals. In this context, dedicated EGS wards may make major improvements in surgically sick patients. Although the importance of EGS is understood, the interest to promote it is limited. This is mainly due to the lack of adequate economic gain, considering especially the shifts and the number of hours of extra-work. Hospital managers are always in difficulty with EGS and all the emergency disciplines due to the continuous drain in funds linked to their activity. As a consequence, investments and long-term plans are always lacking. This is paradoxical because EGS uses and influences a lot of hospital and sanitary systems resources: good plans and organizational efforts would result in an optimized integration of such diffused discipline.

## Main text

During the daily practice of most of the general surgeons around the globe, the evaluation and management of surgical emergencies is one of the main activities. Each one of us started to manage surgical emergencies very early during his/her residency.

The residency formation program is different thorough the world and the various countries but the most complete one would be the one combining traumatic and non-traumatic surgical emergencies together with elective surgical activity in the different fields of surgery. Nowadays, no univocal training/residency plan definition for the emergency general surgeon exists. Years ago, was advocated the necessity of at least, a continent-based training program [4]. At present, however, it has not been realized nor planned.

One of the points during general surgical training is the consideration into which the emergencies are posed. It is a common view that if one surgeon is good in his/her own specialty he/she is capable by definition to manage with surgical emergencies. This presumption is incorrect. Emergency General Surgery is a well-defined surgical specialty and necessitated of a dedicated training and update program in order to be performed at best. As already said EGS was identified as multidisciplinary surgery performed for traumatic and non-traumatic acute conditions during the same admission to the hospital. EGS represents the easiest viable way to provide an affordable and high-quality level of care to emergency surgical and trauma patients [5]. As all well-defined surgical specialties, EGS must have a formation and evaluation project and it is actually a reality thanks to the World Society of Emergency Surgery (WSES).

**Fig. 1:**
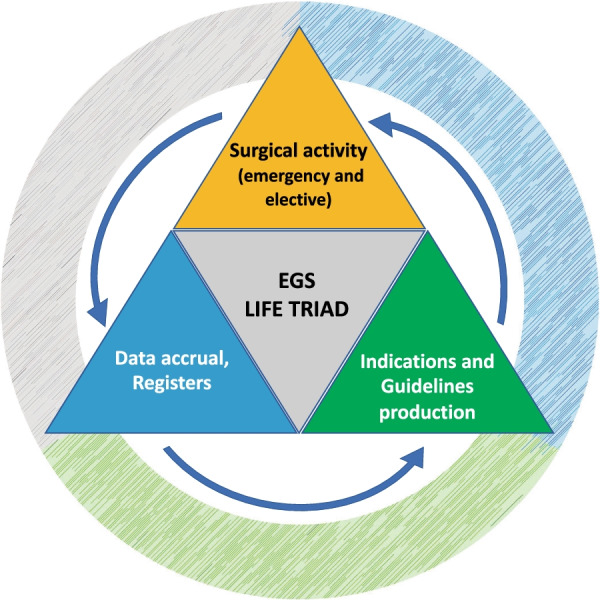
LIFE TRIAD of Emergency General Surgery

As with the critically injured trauma patients, attention to physiological derangement represented by the ***LETHAL triad*** is far more important than definitive surgery in reducing the risk for the patients. Similarly, in EGS the ***LIFE TRIAD*** must be respected and promoted to allow this specialty to be fully effective to grow and to offer the best service to sick patients (Fig. 1).

These three main aspects are as follows:

- Training and continuous surgical activity (emergency and elective)—to maintain and accrue new surgical skills
- Data accrual, registries implementation, and research—to ask questions and produce answers
- Indication and guidelines production and update—for a more universal code of management

*Surgical activity* must be adequate and continuous as must encompass emergency surgical procedures and elective visceral surgical activity. A good emergency general surgeon cannot be fully trained in performing emergency procedures if they are not continuously exposed to elective visceral surgical interventions. This is even more evident if considering the mini-invasive approach and laparoscopic techniques. On the other hand, with fewer open procedures, these skills must be maintained. The necessity to know surgical interventions and their possible variations in order to face emergency situation mandates experience in elective general surgery. The expertise obtained from elective activity allows declining the general surgery to the emergency setting. The EGS sometimes imposes to modify anatomy (temporarily or definitively) to allow the physiology to be restored; the easiest example may be the open abdomen procedures. For these reasons, hospital directors, regional and national healthcare mangers, and providers must warrant enough room for the EGS dept. to perform elective and emergency surgical procedures. This will translate into reduced human and economic costs in emergency patients management.

*Data accrual and registry implementation* are fundamental in producing data to analyze activity and to study large-scale effects of EGS. Limited data accrual is the most limiting defect of many surgical specialties. Examples of effective nation-wide data accrual have been shown by US National Trauma and Emergency Surgery Quality Improvement Programs. They are, however, nation-limited data registries, effective but within the nation in which they are developed and utilized. In EGS randomized trials or complex methodological design studies may result very difficult to realize. For this reason, no high-quality evidence exists in many fields. EGS at present can count on the Web-based International Register of Emergency Surgery and Trauma (WIRES-T) (www.clincalregisters.com) that will overcome this lacking and will allow to perform large-scale analysis including data from different situations and allowing to compare them [6]. WIRES-T is a worldwide diffuse online registry of all the operative and non-operative management of surgical and trauma emergencies. It is free and open to participation. This will give effective and useful answers to the thousands of open questions in EGS. All those who will regularly enter patients and update data will participate in the derived publications. Thanks to this common effort, several high-quality evidence-based guidelines and reviews will emerge and improvements to the existing guidelines will be completed. These data that were previously unable to be captured can now be used more readily as system capacities for data acquisition, storage, and processing are becoming more easily accessible. Greater access to technology can provide EGS clinicians with more data than ever before. New data collection methods can be utilized to address the need for EGS‐specific process and outcome metrics as well as quality improvement programs. Future improvements and developments in big data can inform and guide the further growth of EGS as a new surgical specialty.

*Indications and guidelines production* is a substantial part to diffuse good clinical and surgical practice. Guideline development is only possible through the availability of high-quality data and experts who develop consensus to define the best strategies for specific scenarios where no definitive data may be obtained. Many diseases in fact have been deeply investigated and plenty of literature works exist. A few others, however, due to the paucity of cases and their scattered diffusion, have not been so effectively studied. This is why registries and experts are vital for EGS. The leading societies have the duty and the responsibility to promote expert discussion and production of guidelines. They must offer open and wide options to allow worldwide coming experts to share data, experiences, and opinions in order to obtain the most shared and diffusible guidelines and indications possible.

## Conclusion

The LIFE TRIAD is a concept that must spread and be part of the EGS common practice. As far as it will be the leading light of this great specialty, it will grow and diffuse taking its fruit and effects in each corner of the planet. EGS best practice needs surgeons fully trained to perform general surgical procedures in both elective and emergency settings. They must practice routinely in both fields taking care to accrue data and following the indications and guidelines given by the leading societies in order to provide the best possible care to patients.

## Data Availability

Not applicable.

## Abbreviations

EGS: Emergency General Surgery
WSES: World Society of Emergency Surgery
WIRES-T: Web-based International Register of Emergency Surgery and Trauma
